# Medicare Plan Switching Among Beneficiaries With and Without a History of Cancer

**DOI:** 10.1001/jamanetworkopen.2025.13394

**Published:** 2025-06-03

**Authors:** Shelley A. Jazowski, Emma M. Achola, Lauren Hersch Nicholas, Youngmin Kwon, William A. Wood, Christopher R. Friese, Stacie B. Dusetzina

**Affiliations:** 1 Department of Social Sciences and Health Policy, Wake Forest University School of Medicine, Winston-Salem, North Carolina; 2 Department of Health Policy, Vanderbilt University School of Medicine, Nashville, Tennessee; 3 Division of Geriatrics, Department of Medicine, University of Colorado Anschutz Medical Campus, Aurora; 4 University of Colorado Comprehensive Cancer Center, Aurora; 5 Department of Medicine, University of North Carolina at Chapel Hill School of Medicine, Chapel Hill; 6 Lineberger Comprehensive Cancer Center, University of North Carolina at Chapel Hill, Chapel Hill; 7 University of Michigan School of Nursing, Ann Arbor; 8 Department of Health Management and Policy, University of Michigan School of Public Health, Ann Arbor; 9 Rogel Cancer Center, University of Michigan, Ann Arbor; 10 Vanderbilt-Ingram Cancer Center, Nashville, Tennessee

## Abstract

**Importance:**

The role of supplemental coverage (eg, Medigap) in Medicare enrollment and disenrollment is understudied among beneficiaries with cancer. Understanding the association between initially selecting a supplement and switching plans is necessary to ensure adequate and affordable coverage for beneficiaries with high-cost health conditions.

**Objective:**

To evaluate Medicare plan switching by initial plan selection and history of cancer.

**Design, Setting, and Participants:**

This cohort study used data from the Health and Retirement Study, from 2008 to 2020. Respondents were aged 65 or 66 years at initial Medicare plan selection and completed 2 consecutive surveys. Analyses were conducted from November 2023 to October 2024.

**Exposures:**

Self-reported initial Medicare plan selection (traditional Medicare [TM] without supplemental coverage, TM plus supplemental coverage, or Medicare Advantage [MA]) and history of cancer.

**Main Outcomes and Measures:**

The primary outcome was self-reported Medicare plan switching in the 2 years after initial plan selection. Modified Poisson regression was used to assess the association between initial Medicare plan selection and plan switching. Models adjusted for baseline sociodemographic and health-related characteristics and were stratified by a history of cancer.

**Results:**

Among 2852 respondents aging into Medicare (1113 male [39.02%]), 1511 (52.98%) initially selected TM plus supplemental coverage and 358 (12.55%) reported a history of cancer. Less than one-third of beneficiaries (786 beneficiaries without a history of cancer [31.52%] and 106 beneficiaries with a history of cancer [29.61%]) switched coverage in the 2 years after initial plan selection. Most beneficiaries switched from TM without supplemental coverage to benefits with greater financial protections; 131 of 227 beneficiaries without a history of cancer (57.71%) switched to MA, and 18 of 27 beneficiaries with a history of cancer (66.67%) switched to TM plus supplemental coverage. Among beneficiaries without a history of cancer, initial selection of MA (adjusted risk ratio [aRR], 0.55; 95% confidence limit [CL], 0.47-0.64) or TM plus supplemental coverage (aRR, 0.63; 95% CL, 0.55-0.72) was associated with a lower probability of switching coverage compared with initial selection of TM without supplemental coverage. Findings were similar for beneficiaries with a history of cancer.

**Conclusions and Relevance:**

In this cohort study of older adults with and without a history of cancer, initial selection of MA or TM plus supplemental coverage was associated with a low likelihood of switching coverage. Given that benefits with greater financial protections may meet beneficiaries’ evolving needs and preferences, policymakers should consider improving the adequacy of TM.

## Key Points

**Question:**

Does Medicare plan switching differ by initial type of Medicare coverage and history of cancer?

**Findings:**

In this cohort study of 2852 older adults, 31.52% without a history of cancer and 29.61% with a history of cancer switched Medicare coverage in the 2 years following initial plan selection. Initial choice of Medicare Advantage or traditional Medicare plus supplemental coverage was associated with a low likelihood of plan switching among respondents with and without a history of cancer.

**Meaning:**

These findings suggest that Medicare coverage with greater financial protections may better meet the financial and health needs of older adults; as such, policymakers should consider improving the adequacy of traditional Medicare coverage.

## Teaser

This cohort study evaluates Medicare plan switching by initial plan selection and history of cancer.

## Introduction

Nearly two-thirds of Medicare beneficiaries have reported that their coverage fully meets their expectations.^1,2^ However, a recent national survey^2^ has found that 15% of Medicare beneficiaries changed their coverage in the past 2 years and another 6% wanted to but had not yet changed their coverage. Beneficiaries often switch between Medicare Advantage (MA) plans or from MA to traditional Medicare (TM).^2,3,4^ Patterns of switching Medicare coverage are less clear among beneficiaries with cancer. Early studies^5,6^ have found no significant differences in MA disenrollment between beneficiaries with and without cancer. Conversely, contemporary studies^7,8^ have observed a high probability of switching from MA to TM in the year following a cancer diagnosis. Although the rapid growth in MA enrollment^7,8^ and the implementation of lock-in provisions^9^ may explain inconsistencies in findings, these studies provide an incomplete understanding of Medicare enrollment and switching. By predominantly focusing on administrative data, these studies were unable to account for supplemental coverage among beneficiaries who originally enrolled in TM or switched from MA to TM. Most TM beneficiaries are enrolled in supplemental coverage (eg, Medigap or retiree health benefits),^10^ which limits their financial exposure for necessary inpatient and medical services.^10,11,12^ Given both the high probability of patients with cancer initially selecting TM plus supplemental coverage^11^ and that affordability of care is a key consideration when switching coverage,^13,14,15^ our objective was to examine switching by initial Medicare plan choice and history of cancer.

## Methods

This cohort study was approved by the Vanderbilt University Medical Center institutional review board. In accordance with 45 CFR §46, informed consent was not required because the data are publicly available and deidentified. This study followed the Strengthening the Reporting of Observational Studies in Epidemiology (STROBE) reporting guidelines.

### Study Population

We used biennial data (2008-2020) from the Health and Retirement Study^16^ to identify respondents who initially selected Medicare coverage at age 65 or 66 years and completed 2 consecutive surveys (1 baseline survey to measure initial plan selection and history of cancer and 1 subsequent survey to measure switching) (eFigure in Supplement 1). Since our focus was the selection and switching of Medicare coverage, we excluded individuals who were enrolled in Medicare before age eligibility,^11,12^ were dually eligible for Medicaid or enrolled in military health plans at initial plan selection,^11,12^ or disenrolled from Medicare following initial plan selection (eg, switched exclusively to commercial coverage).

### Independent Variables

The main independent variables were self-reported initial Medicare coverage and history of cancer. Medicare coverage was defined as selecting 1 of 3 mutually exclusive categories: TM without supplemental coverage, TM plus supplemental coverage, or MA.^11,12^ Beneficiaries were categorized as having a history of cancer if they responded affirmatively to the following question at the time of initial plan selection: “Has a doctor ever told you that you have cancer or a malignant tumor, excluding minor skin cancer?”

### Outcome

The primary outcome was self-reported switching in the 2 years following initial Medicare plan selection. If Medicare coverage in this time period (eg, MA) differed from initial plan selection (eg, TM without supplemental coverage), then beneficiaries were categorized as having switched coverage.

### Covariates

On the basis of prior evidence of their associations with Medicare enrollment decisions,^2,8,11^ covariates included the following self-reported baseline sociodemographic and health-related characteristics at the time of initial Medicare plan selection: sex, race (White, Black, or other [ie, American Indian, Alaska Native, Asian, Native Hawaiian, and Pacific Islander]), Hispanic ethnicity, marital or partnered status, educational attainment (high school education or less vs above high school education),^11,12^ employment status, US Census region, quartiles of wealth (eg, real estate and investments), quartiles of out-of-pocket spending (beneficiaries’ portion of costs for hospital stays, nursing home stays, outpatient surgeries, physician visits, dentist visits, home health care, special services, and prescription medications), year of initial plan selection, overall health status (fair or poor vs good, very good, or excellent),^12^ comorbid conditions (sum of diagnoses of hypertension, diabetes, stroke, arthritis, lung disease, heart condition, cognitive impairment, and psychological or emotional issues),^11,12^ functional limitations (sum of activities of daily living and instrumental activities of daily living),^11^ and current smoking status.

### Statistical Analysis

We used modified Poisson regression with robust error variance^17^ to estimate the likelihood of switching from initial Medicare coverage. The model adjusted for baseline sociodemographic and health-related factors and was stratified by a history of cancer. We report unweighted primary estimates since survey-weighted estimates may be unstable for measures with a limited number of observations^18^; however, we applied respondent-level survey weights in a sensitivity analysis. We also conducted sensitivity analyses that examined plan switching among beneficiaries who may have postponed Medicare enrollment until retirement (≤75 years of age)^11,12^; excluded individuals who reported a cancer diagnosis after initial Medicare plan selection^12^; and estimated whether a respondent ever switched Medicare coverage (in any survey following the baseline survey). Analyses were conducted from November 2023 to October 2024 using SAS Studio release 9.4 (SAS Institute). Statistical tests were 2-sided, and *P* < .05 denoted statistical significance.

## Results

### Cohort Characteristics

Of the 2852 older adults aging into Medicare (1113 male [39.02%]), 1511 (52.98%) initially selected TM plus supplemental coverage and 358 (12.55%) reported a history of cancer (Table 1). Sociodemographic and health-related characteristics were similar between beneficiaries with and without a history of cancer. However, beneficiaries with a history of cancer were more likely to identify as White (301 beneficiaries [84.08%] vs 1953 beneficiaries [78.31%]), have above a high school education (167 beneficiaries [46.65%] vs 941 beneficiaries [37.73%]), and report multiple comorbid conditions (225 beneficiaries [62.85%] vs 1413 beneficiaries [56.66%]) compared with their counterparts without a history of cancer.

**Table 1.** Baseline Self-Reported Sociodemographic and Health-Related Characteristics of Respondents Aging Into Medicare

Characteristic	Respondents, No. (%)			*P* value
	Total (N = 2852)	With a history of cancer (n = 358)	Without a history of cancer (n = 2494)	
Initial Medicare coverage				
Medicare Advantage	806 (28.26)	102 (28.49)	704 (28.23)	.004
Traditional Medicare plus supplemental coverage	1511 (52.98)	211 (58.94)	1300 (52.13)	
Traditional Medicare without supplemental coverage	535 (18.76)	45 (12.57)	490 (19.65)	
Sex				
Male	1113 (39.02)	133 (37.15)	980 (39.29)	.44
Female	1739 (60.97)	225 (62.85)	1514 (60.71)	
Race				
Black	431 (15.11)	44 (12.29)	387 (15.52)	.03
White	2254 (79.03)	301 (84.08)	1953 (78.31)	
Other^a^	167 (5.86)	13 (3.63)	154 (6.17)	
Hispanic ethnicity				
Yes	250 (8.77)	22 (6.15)	228 (9.14)	.06
No	2602 (91.23)	336 (93.84)	2266 (90.86)	
Married or partnered				
Yes	2130 (74.68)	276 (77.09)	1854 (74.34)	.26
No	722 (25.32)	82 (22.91)	640 (25.66)	
Education				
High school or less	1108 (38.85)	191 (53.35)	1553 (62.27)	.001
Above high school	1744 (61.15)	167 (46.65)	941 (37.73)	
Employed				
Yes	1037 (36.36)	127 (35.47)	910 (36.49)	.71
No	1815 (63.64)	231 (64.53)	1584 (63.51)	
US Census region				
Northeast	375 (13.15)	46 (12.85)	329 (13.19)	.29
Midwest	743 (26.05)	81 (22.63)	662 (26.54)	
South	1180 (41.37)	151 (42.18)	1029 (41.26)	
West	554 (19.42)	80 (22.35)	474 (19.01)	
Wealth quartile, $^b^				
First, <84 000	695 (24.37)	91 (25.42)	604 (24.22)	.48
Second, 84 000-295 000	710 (24.89)	81 (22.63)	629 (25.22)	
Third, 295 001-728 500	726 (25.46)	86 (24.02)	640 (25.66)	
Fourth, >728 500	721 (25.28)	100 (27.93)	621 (24.90)	
Out-of-pocket spending quartile, $^c^				
First, <571	713 (25.00)	66 (18.44)	647 (25.94)	<.001
Second, 572-1645	713 (25.00)	80 (22.35)	633 (25.38)	
Third, 1666-3869	714 (25.04)	95 (26.54)	619 (24.82)	
Fourth, >3869	712 (24.96)	117 (32.68)	595 (23.86)	
Initial Medicare enrollment year				
2008	568 (19.92)	65 (18.16)	503 (20.17)	.04
2010	438 (15.36)	60 (16.76)	378 (15.16)	
2012	427 (14.97)	60 (16.76)	367 (14.72)	
2014	481 (16.87)	72 (20.11)	409 (16.40)	
2016	516 (18.09)	45 (12.57)	471 (18.89)	
2018	422 (14.80)	56 (15.64)	366 (14.68)	
Overall health				
Excellent, very good, or good	442 (15.50)	289 (80.73)	2121 (85.04)	.03
Fair or poor	2410 (84.50)	69 (19.27)	373 (14.96)	
Comorbid conditions^d^				
0	407 (14.27)	38 (10.61)	369 (14.80)	.04
1	807 (28.30)	95 (26.54)	712 (28.55)	
≥2	1638 (57.43)	225 (62.85)	1413 (56.66)	
Functional limitations^e^				
0	1150 (40.32)	138 (38.55)	1012 (40.58)	.41
1	582 (20.41)	68 (18.99)	514 (20.61)	
≥2	1120 (39.27)	152 (42.46)	968 (38.81)	
Current smoker				
Yes	317 (11.12)	31 (8.66)	286 (11.47)	.11
No	2535 (88.88)	327 (91.34)	2208 (88.53)	

^a^ Other race included American Indian, Alaska Native, Asian, Native Hawaiian, and Pacific Islander.

^b^ Self-reported quartiles of wealth and assets were defined using the total wealth RAND variable (sum value of residences, vehicles, investments, and bank accounts or savings less mortgages, loans, and debts).

^c^ Out-of-pocket spending included the beneficiary’s portion of costs for hospital stays, nursing home stays, outpatient surgeries, physician visits, dentist visits, home health care, special services, and prescription medications.

^d^ Self-reported comorbidities included hypertension, diabetes, stroke, arthritis, lung disease, heart condition, cognitive impairment, and psychological or emotional issues.

^e^ Self-reported functional limitations included activities of daily living and instrumental activities of daily living.

### Switching From Initial Medicare Coverage

Less than one-third of beneficiaries (786 beneficiaries without a history of cancer [31.52%]; 106 beneficiaries with a history of cancer [29.61%]) switched Medicare coverage in the 2 years after initial plan selection (eTable 1 in Supplement 1). Reports of plan switching were most common among respondents who initially selected TM without supplemental coverage (227 beneficiaries without a history of cancer [46.33%]; 27 beneficiaries with a history of cancer [60.00%]). Among respondents who switched from TM without supplemental coverage, 57.71% of those without a history of cancer (131 of 227 beneficiaries) opted for MA, whereas 66.67% of those with a history of cancer (18 of 27 beneficiaries) selected TM plus supplemental coverage (Figure).

**Figure.**
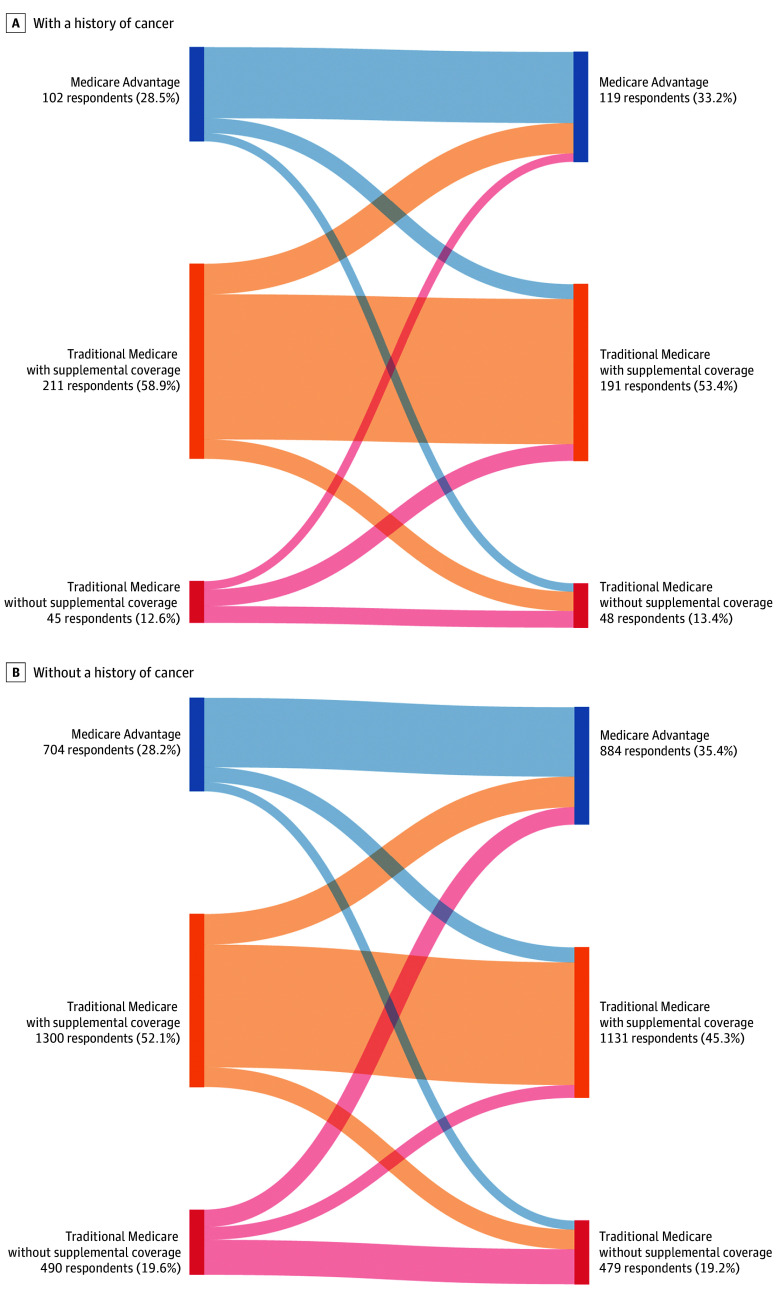
Switching From Initial Medicare Coverage by a History of Cancer Figure was created with online tool, SankeyMATIC. Figure displays Medicare plan switching in the 2 years (surveys are administered biennially) following initial plan selection. Panel A shows respondents with a history of cancer, and panel B shows respondents without a history of cancer.

A history of cancer was not associated with switching from initial Medicare coverage (adjusted risk ratio [aRR], 0.98; 95% confidence limit [CL], 0.83-1.16) (eTable 2 in Supplement 1). Among beneficiaries without a history of cancer, those initially choosing MA or TM plus supplemental coverage had a 45% (aRR, 0.55; 95% CL, 0.47-0.64) or 37% (aRR, 0.63; 95% CL, 0.55-0.72) lower likelihood of switching plans, respectively, compared with their counterparts who initially selected TM without supplemental coverage (Table 2). Findings were similar for beneficiaries with a history of cancer.

**Table 2.** Association of Initial Plan Selection and Switching Medicare Coverage

Variable	Adjusted RR (95% CL)^a^		
	Entire study population	Respondents with a history of cancer	Respondents without a history of cancer
Medicare Advantage	0.54 (0.46-0.63)	0.49 (0.31-0.77)	0.55 (0.47-0.64)
Traditional Medicare plus supplemental coverage	0.63 (0.55-0.71)	0.58 (0.41-0.82)	0.63 (0.55-0.72)
Traditional Medicare without supplemental coverage	1 [Reference]	1 [Reference]	1 [Reference]

Abbreviations: CL, confidence limit; RR, risk ratio.

^a^ Models adjusted for baseline sociodemographic and health-related characteristics. Full model results are shown in eTable 2 in Supplement 1.

### Sensitivity Analysis

We conducted sensitivity analyses that applied survey weights, expanded beneficiaries’ ages at initial plan selection, excluded reports of cancer diagnoses after initial plan selection, and estimated ever switching coverage. The findings were similar to those of our primary analysis (eTable 3 in Supplement 1).

## Discussion

To our knowledge, this cohort study is one of the first to account for supplemental coverage when examining Medicare plan switching among beneficiaries with and without a cancer diagnosis. Among beneficiaries with and without a history of cancer, we observed low rates of switching from TM plus supplemental coverage, but high rates of switching from TM without supplemental coverage. High cost-sharing (20% coinsurance)^10,11^ may have impacted the affordability of necessary care and prompted TM beneficiaries to enroll in supplemental coverage or switch to an MA plan with out-of-pocket maximums.

Consistent with prior research,^5,6,15^ we did not observe an association between a history of cancer and switching Medicare coverage. Beneficiaries with a history of cancer may have anticipated their health care needs when enrolling in Medicare and, thus, were satisfied with their initial coverage.^10,11^ Specifically, most beneficiaries with a history of cancer initially selected TM plus supplemental coverage, which would not only aid with cancer-related expenses (eg, average of $41 800 and $5300 for initial and continuing medical care, respectively)^19^ but also allow for visits with any health care practitioner (eg, specialist) who accepts Medicare.^11^

Given the switching patterns of beneficiaries with and without a history of cancer who initially selected TM without supplemental coverage, policymakers should consider improving TM coverage, including expanding the eligibility for and comprehensiveness of Medicare Savings Programs and extending consumer protections for supplemental coverage. Together, these actions could support the 3.2 million TM beneficiaries^10^ with and without a history of cancer who lack supplemental coverage and are at increased risk of financial burden.

### Limitations

Our study had several limitations. First, we were unable to discern the reasons for switching Medicare coverage. Future research is needed to understand beneficiaries’ rationale and preferences for initially selecting and switching Medicare coverage. Second, although data were self-reported, data collection methods minimize the extent of biases (eg, recall),^16^ and many variables have demonstrated high validity compared with administrative or longitudinal survey data.^20,21^ Third, although the sample size of beneficiaries with a history of cancer may have impacted comparisons across types of Medicare coverage, we addressed limited samples size by applying respondent-level survey weights in a sensitivity analysis with results similar to our primary findings. Fourth, owing to the structure of the Health and Retirement Study, we were only able to examine switching in the 2 years following initial plan selection and, thus, may have underestimated the prevalence of switching among beneficiaries with and without a history of cancer. As the time period in which switching is assessed may potentially explain inconsistencies with recent research,^7,8^ future studies are needed to understand plan switching in the year immediately following diagnosis. Fifth, we lacked information regarding timing and severity of a cancer diagnosis, both of which could have influenced plan switching.

## Conclusions

In this cohort study of older adults with and without a history of cancer, we found that those who initially selected either MA or TM plus supplemental coverage had a lower likelihood of switching Medicare coverage compared with their counterparts who initially chose TM without supplemental coverage. Policymakers should consider improving the adequacy of TM coverage.

## Notes

Supplement 1. **eFigure**. Study Flow Diagram **eTable 1.** Medicare Plan Switching by History of Cancer **eTable 2.** Self-reported Sociodemographic and Health-related Characteristics Associated with Switching from Initial Medicare Coverage **eTable 3.** Sensitivity Analyses of Medicare Plan Switching by History of Cancer

Supplement 2.
Data Sharing Statement
