# Supplementary material for: Medicare Plan Switching Among Beneficiaries With and Without a History of Cancer
Source: JAMA Netw Open. 2025 Jun 3;8(6):e2513394. doi: 10.1001/jamanetworkopen.2025.13394 (PMC12134947; doi:10.1001/jamanetworkopen.2025.13394)
Supplement: Supplement 1. — eFigure. Study Flow Diagram eTable 1. Medicare Plan Switching by History of Cancer eTable 2. Self-reported Sociodemographic and Health-related Characteristics Associated with Switching from Initial Medicare Coverage eTable 3. Sensitivity Analyses of Medicare Plan Switching by History of Cancer [file jamanetwopen-e2513394-s001.pdf]

Supplemental Online Content

Jazowski SA, Achola EM, Nicholas LH, et al. Medicare plan switching among beneficiaries with and without a history of cancer. *JAMA Netw Open*. 2025;8(6):e2513394. doi:10.1001/jamanetworkopen.2025.13394

eFigure. Study Flow Diagram

eTable 1. Medicare Plan Switching by History of Cancer

eTable 2. Self-reported Sociodemographic and Health-related Characteristics Associated with Switching from Initial Medicare Coverage

eTable 3. Sensitivity Analyses of Medicare Plan Switching by History of Cancer

This supplemental material has been provided by the authors to give readers additional information about their work.

© 2025 Jazowski SA et al. *JAMA Network Open*.

eFigure. Study Flow Diagram

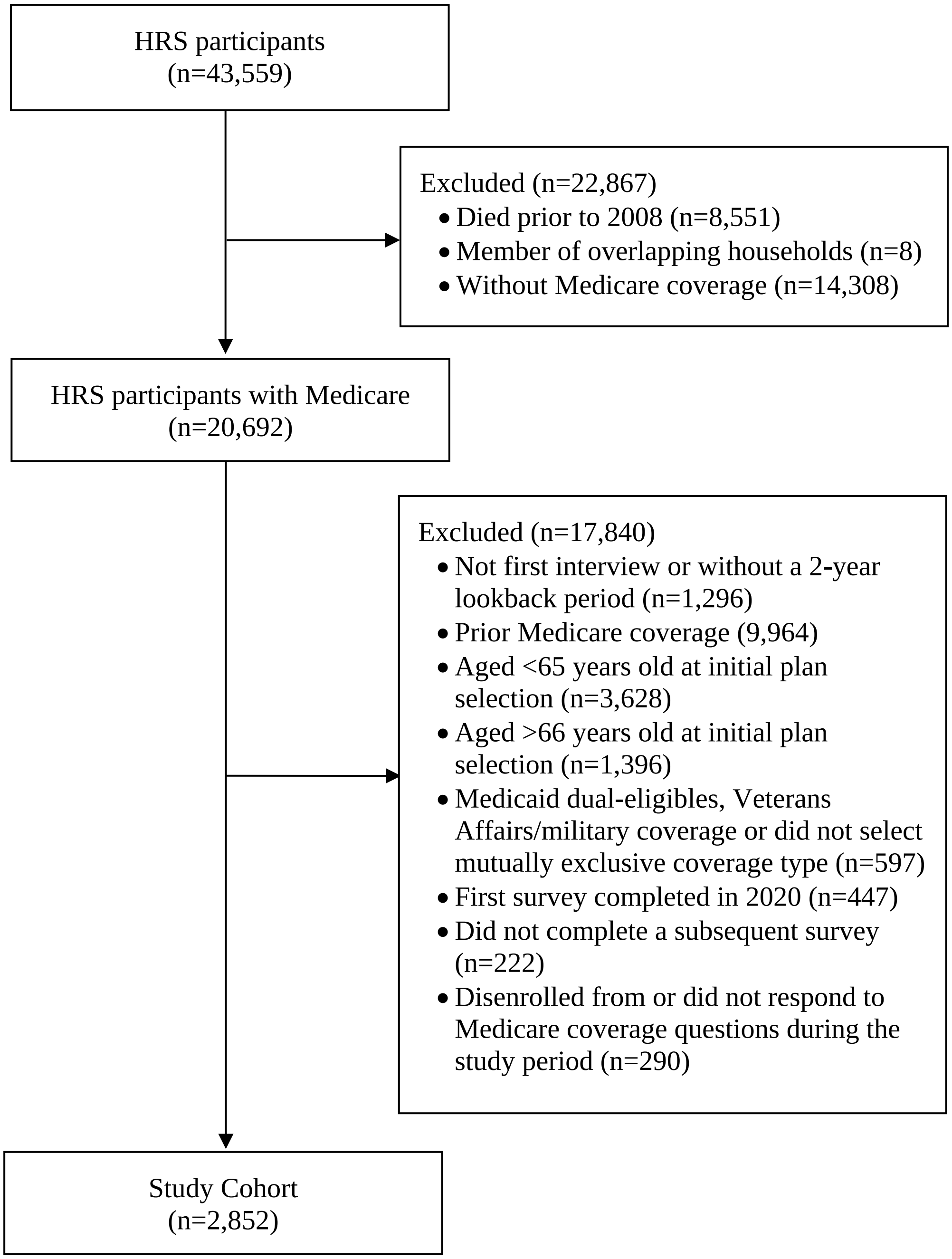

Abbreviations: HRS; Health and Retirement Study

© 2025 Jazowski SA et al. *JAMA Network Open*.

eTable 1. Medicare Plan Switching by History of Cancer

Switching Among the Study Population			
	MA	TM with supplemental coverage	TM without supplemental coverage
MA (n=806)		129	77
TM with supplemental coverage (n=1,511)	263		169
TM without supplemental coverage (n=535)	140	114	
**Switching Among Respondents with a History of Cancer**			
	MA	TM with supplemental coverage	TM without supplemental coverage
MA (n=102)		16	9
TM with supplemental coverage (n=211)	33		21
TM without supplemental coverage (n=45)	9	18	
**Switching Among Respondents without a History of Cancer**			
	MA	TM with supplemental coverage	TM without supplemental coverage
MA (n=704)		113	68
TM with supplemental coverage (n=1,300)	230		148
TM without supplemental coverage (n=490)	131	96	

Abbreviations: MA, Medicare Advantage; TM, traditional Medicare

© 2025 Jazowski SA et al. *JAMA Network Open*.

eTable 2. Self-reported Sociodemographic and Health-related Characteristics Associated with Switching from Initial Medicare Coverage

	Study Population	Respondents with a History of Cancer	Respondents without a History of Cancer
	**Adjusted Risk Ratios (95% Confidence Limits)**		
History of cancer			
Yes	0.98 (0.83-1.16)		
No	Ref		
Initial Medicare coverage			
Medicare Advantage	0.54 (0.46-0.63)	0.49 (0.31-0.77)	0.55 (0.47-0.64)
Traditional Medicare plus supplemental coverage	0.63 (0.55-0.71)	0.58 (0.41-0.82)	0.63 (0.55-0.72)
Traditional Medicare without supplemental coverage	Ref	Ref	Ref
Sex			
Male	Ref	Ref	Ref
Female	0.93 (0.83-1.04)	0.82 (0.61-1.11)	0.94 (0.84-1.06)
Race			
Black	1.15 (1.00-1.33)	1.50 (1.00-2.23)	1.13 (0.97-1.32)
White	Ref	Ref	Ref
Other[a]	1.06 (0.83-1.36)	1.54 (0.62-3.81)	1.03 (0.80-1.34)
Hispanic ethnicity			
Yes	0.89 (0.72-1.11)	0.69 (0.32-1.49)	0.90 (0.72-1.14)
No	Ref	Ref	Ref
Married/partnered			
Yes	Ref	Ref	Ref
No	0.94 (0.82-1.07)	1.00 (0.69-1.46)	0.92 (0.80-1.06)
Education			
High school or less	1.07 (0.95-1.21)	1.20 (0.86-1.67)	1.06 (0.93-1.21)
Above high school	Ref	Ref	Ref
Employment			
Yes	Ref	Ref	Ref
No	0.96 (0.86-1.07)	0.89 (0.64-1.22)	0.97 (0.86-1.10)
Census region			
Northeast	0.93 (0.78-1.11)	0.53 (0.25-1.13)	0.99 (0.82-1.18)
Midwest	0.96 (0.84-1.10)	1.26 (0.85-1.88)	0.93 (0.81-1.08)
South	Ref	Ref	Ref
West	1.02 (0.87-1.19)	1.21 (0.80-1.83)	0.98 (0.83-1.16)
Wealth[b]			
Quartile 1: <$84,000	1.04 (0.86-1.26)	1.39 (0.86-2.24)	0.96 (0.79-1.18)
Quartile 2: $84,000-$295,000	1.19 (1.01-1.40)	1.38 (0.85-2.23)	1.15 (0.97-1.37)
Quartile 3: $295,001-$728,500	1.01 (0.86-1.19)	0.88 (0.51-1.51)	1.01 (0.85-1.19)
Quartile 4: >$728,500	Ref	Ref	Ref
Out-of-pocket spending[c]			

© 2025 Jazowski SA et al. *JAMA Network Open*.

Quartile1: <$571	0.86 (0.74-1.01)	0.73 (0.47-1.13)	0.89 (0.75-1.05)
Quartile 2: $572-$1,645	0.89 (0.77-1.04)	0.72 (0.45-1.16)	0.95 (0.80-1.11)
Quartile 3: $1,666-$3,869	0.85 (0.73-0.99)	0.63 (0.41-0.98)	0.90 (0.76-1.06)
Quartile 4: >$3,869	Ref	Ref	Ref
Initial Medicare enrollment year			
2008	Ref	Ref	Ref
2010	0.93 (0.77-1.13)	1.72 (0.95-3.14)	0.84 (0.68-1.04)
2012	1.05 (0.87-1.26)	1.17 (0.61-2.25)	1.04 (0.86-1.26)
2014	0.99 (0.82-1.19)	1.50 (0.80-2.81)	0.94 (0.77-1.15)
2016	1.12 (0.94-1.34)	1.15 (0.55-2.38)	1.11 (0.93-1.34)
2018	1.30 (1.09-1.55)	2.29 (1.27-4.15)	1.22 (1.01-1.47)
Overall health			
Excellent, very good, or good	Ref	Ref	Ref
Fair or poor	1.07 (0.92-1.25)	0.83 (0.56-1.23)	1.11 (0.95-1.31)
Comorbid conditions[d]			
0	Ref	Ref	Ref
1	1.11 (0.93-1.33)	0.68 (0.40-1.15)	1.16 (0.95-1.41)
≥2	1.02 (0.85-1.23)	0.57 (0.35-0.93)	1.09 (0.90-1.33)
Functional limitations[e]			
0	Ref	Ref	Ref
1	1.10 (0.95-1.27)	1.99 (1.29-3.09)	1.01 (0.87-1.18)
≥2	0.98 (0.85-1.12)	1.60 (1.05-2.45)	0.92 (0.98-1.06)
Current smoker			
Yes	1.10 (0.93-1.29)	1.46 (0.93-2.29)	1.10 (0.92-1.31)
No	Ref	Ref	Ref

[a] Other race included American Indian, Alaskan Native, Asian, Native Hawaiian, and Pacific Islander.
[b] Self-reported quartiles of wealth and assets were defined using the total wealth RAND variable (sum value of residences, vehicles, investments, bank accounts/savings less mortgages, loans, and debts).
[c] Out-of-pocket spending included the beneficiary's portion of costs for hospital stays, nursing home stays, outpatient surgeries, physician visits, dentist visits, home health care, special services, and prescription medications.
[d] Self-reported comorbidities included hypertension, diabetes, stroke, arthritis, lung disease, heart condition, cognitive impairment, psychological or emotional issues.
[e] Self-reported functional limitations included activities of daily living and instrumental activities of daily living.

© 2025 Jazowski SA et al. *JAMA Network Open*.

eTable 3. Sensitivity Analyses of Medicare Plan Switching by History of Cancer

	**Study Population**	**Respondents with a History of Cancer**	**Respondents without a History of Cancer**
Survey-weighted analysis[a]			
Initial Medicare coverage			
Medicare Advantage	0.57 (0.48-0.68)	0.65 (0.38-1.12)	0.57 (0.47-0.69)
Traditional Medicare plus supplemental coverage	0.59 (0.50-0.68)	0.64 (0.42-1.00)	0.59 (0.50-0.69)
Traditional Medicare without supplemental coverage	Ref	Ref	Ref
Expanded beneficiary age at initial plan selection[b]			
Initial Medicare coverage			
Medicare Advantage	0.55 (0.48-0.62)	0.52 (0.36-0.75)	0.55 (0.47-0.63)
Traditional Medicare plus supplemental coverage	0.64 (0.60-0.72)	0.58 (0.42-0.80)	0.65 (0.57-0.73)
Traditional Medicare without supplemental coverage	Ref	Ref	Ref
Excluded respondents with a cancer diagnosis after initial plan selection[c]			
Initial Medicare coverage			
Medicare Advantage	0.54 (0.47-0.63)	0.49 (0.31-0.77)	0.55 (0.47-0.65)
Traditional Medicare plus supplemental coverage	0.63 (0.55-0.72)	0.58 (0.41-0.82)	0.64 (0.56-0.74)
Traditional Medicare without supplemental coverage	Ref	Ref	Ref
Ever switched Medicare coverage[d]			
Initial Medicare coverage			
Medicare Advantage	0.57 (0.52-0.64)	0.51 (0.38-0.71)	0.58 (0.52-0.64)
Traditional Medicare plus supplemental coverage	0.81 (0.75-0.87)	0.72 (0.57-0.91)	0.82 (0.76-0.88)
Traditional Medicare without supplemental coverage	Ref	Ref	Ref

[a] Analysis applied respondent-level survey weights.

[b] Analysis included beneficiaries who were 65 to 75 years of age at initial Medicare plan selection. 451 respondents with a history of cancer and 3,102 respondents without a history of cancer were included in the analysis.

[c] Analysis excluded beneficiaries who reported a cancer diagnosis in the survey wave after initial Medicare plan selection (n=75). 358 respondents with a history of cancer and 2,419 respondents without a history of cancer were included in the analysis.

[d] Analysis assessed switching Medicare coverage in any survey wave following initial Medicare plan selection.

© 2025 Jazowski SA et al. *JAMA Network Open*.
