# Supplementary material for: Medicare Plan Switching Among Beneficiaries With and Without a History of Cancer
Source: JAMA Netw Open. 2025 Jun 3;8(6):e2513394. doi: 10.1001/jamanetworkopen.2025.13394 (PMC12134947; doi:10.1001/jamanetworkopen.2025.13394)
Supplement: Supplement 2. — Data Sharing Statement [file jamanetwopen-e2513394-s002.pdf]

Data Sharing Statement

Jazowski. Medicare Plan Switching Among Beneficiaries With and Without a History of Cancer. *JAMA Netw Open*. Published June 03, 2025. doi:10.1001/jamanetworkopen.2025.13394

Data
Data available: No

Additional Information
Explanation for why data not available: The data used are available via application to the Health and Retirement Study.
